# Identifying Mouse Autoimmune Uveitis from Fundus Photographs Using Deep Learning

**DOI:** 10.1167/tvst.9.2.59

**Published:** 2020-12-02

**Authors:** Jian Sun, Xiaoqin Huang, Charles Egwuagu, Youakim Badr, Stephen Charles Dryden, Brian Thomas Fowler, Siamak Yousefi

**Affiliations:** 1 Molecular Immunology Section, Laboratory of Immunology, National Eye Institute, National Institutes of Health, Bethesda, MD, USA; 2 The Pennsylvania State University Great Valley, Malvern, PA, USA; 3 University of Tennessee Health Science Center, Memphis, TN, USA

**Keywords:** uveitis, deep learning, convolution neural network, fundus image, artificial intelligence

## Abstract

**Purpose:**

To develop a deep learning model for objective evaluation of experimental autoimmune uveitis (EAU), the animal model of posterior uveitis that reveals its essential pathological features via fundus photographs.

**Methods:**

We developed a deep learning construct to identify uveitis using reference mouse fundus images and further categorized the severity levels of disease into mild and severe EAU. We evaluated the performance of the model using the area under the receiver operating characteristic curve (AUC) and confusion matrices. We further assessed the clinical relevance of the model by visualizing the principal components of features at different layers and through the use of gradient-weighted class activation maps, which presented retinal regions having the most significant influence on the model.

**Results:**

Our model was trained, validated, and tested on 1500 fundus images (training, 1200; validation, 150; testing, 150) and achieved an average AUC of 0.98 for identifying the normal, trace (small and local lesions), and disease classes (large and spreading lesions). The AUCs of the model using an independent subset with 180 images were 1.00 (95% confidence interval [CI], 0.99–1.00), 0.97 (95% CI, 0.94–0.99), and 0.96 (95% CI, 0.90–1.00) for the normal, trace and disease classes, respectively.

**Conclusions:**

The proposed deep learning model is able to identify three severity levels of EAU with high accuracy. The model also achieved high accuracy on independent validation subsets, reflecting a substantial degree of generalizability.

**Translational Relevance:**

The proposed model represents an important new tool for use in animal medical research and provides a step toward clinical uveitis identification in clinical practice.

## Introduction

Posterior uveitis represents a diverse group of potentially sight-threatening intraocular inflammatory diseases of infectious or autoimmune etiology that includes, but is not limited to, Behçet's disease, birdshot retinochoroidopathy, Vogt–Koyanagi–Harada disease, sympathetic ophthalmia, and ocular sarcoidosis. Posterior uveitis accounts for more than 10% of severe visual handicaps in the United States.^1^ Experimental autoimmune uveitis (EAU) is an animal disease that shares essential pathological features with human uveitis and is widely used as the animal model for uveitis. It is a predominantly T-cell-mediated intraocular inflammatory disease induced in susceptible species by active immunization with retinal proteins or their peptides. EAU is a valuable model for studying the mechanisms of human uveitis and for evaluating the efficacy of new therapies and diagnostic methodologies.^2^ However, clinical assessment and evaluation of disease severity are subject to substantial inter- and intra-observer variability.^3^ For example, some mild pathological changes are often misdiagnosed by human clinicians.^4^ However, accurate detection and monitoring of disease severity are crucial for tailoring medical therapy to avoid performing therapeutic interventions that carry risks of significant adverse ocular and systemic side effects. Development of automated tools to objectively and accurately characterize uveitis-induced pathological changes is an unmet need.

Artificial intelligence (AI) has emerged as a powerful method to assist humans in routine and complex tasks in science and medicine.^5^^,^^6^^,^^7^^–^^10^ In recent years, deep learning, which is a subfield of AI, has been successfully applied to the diagnosis of human retinal diseases, including diabetic retinopathy,^11^^,^^12^ glaucoma,^13^^,^^14^ and age-related macular degeneration.^15^^–^^17^ Several deep learning-based models have been developed to recognize a variety of retinal conditions. Son et al.^4^ recently reported a series of models that identify 12 types of retina lesions based on analysis of fundus images with accuracy comparable to that of experienced ophthalmologists. Thus, deep learning models such as convolutional neural networks (CNNs) hold promise for providing unbiased detection and classification systems for retinal diseases.^9^

Although the CNNs that form the basis of deep learning in human clinical settings have been well developed, few studies have applied deep learning to veterinary ophthalmology or laboratory research. We hypothesize that disease features in mouse models could also be effectively analyzed and scored automatically by deep learning and could potentially be generalized for further implementation in animal-based clinical studies and practice.

In this study, we developed a CNN model to provide unbiased characterization of uveitis in mice based on analysis of mouse fundus images. We first employed transfer learning techniques to reuse pretrained models in recognizing general features in images, and then we adapted the construct to specifically learn EAU-induced signs in fundus images. Our results demonstrated remarkable accuracy in both testing and independent validation subsets.

## Methods

### Dataset of Experimental Autoimmune Uveitis

**Figure 1.**
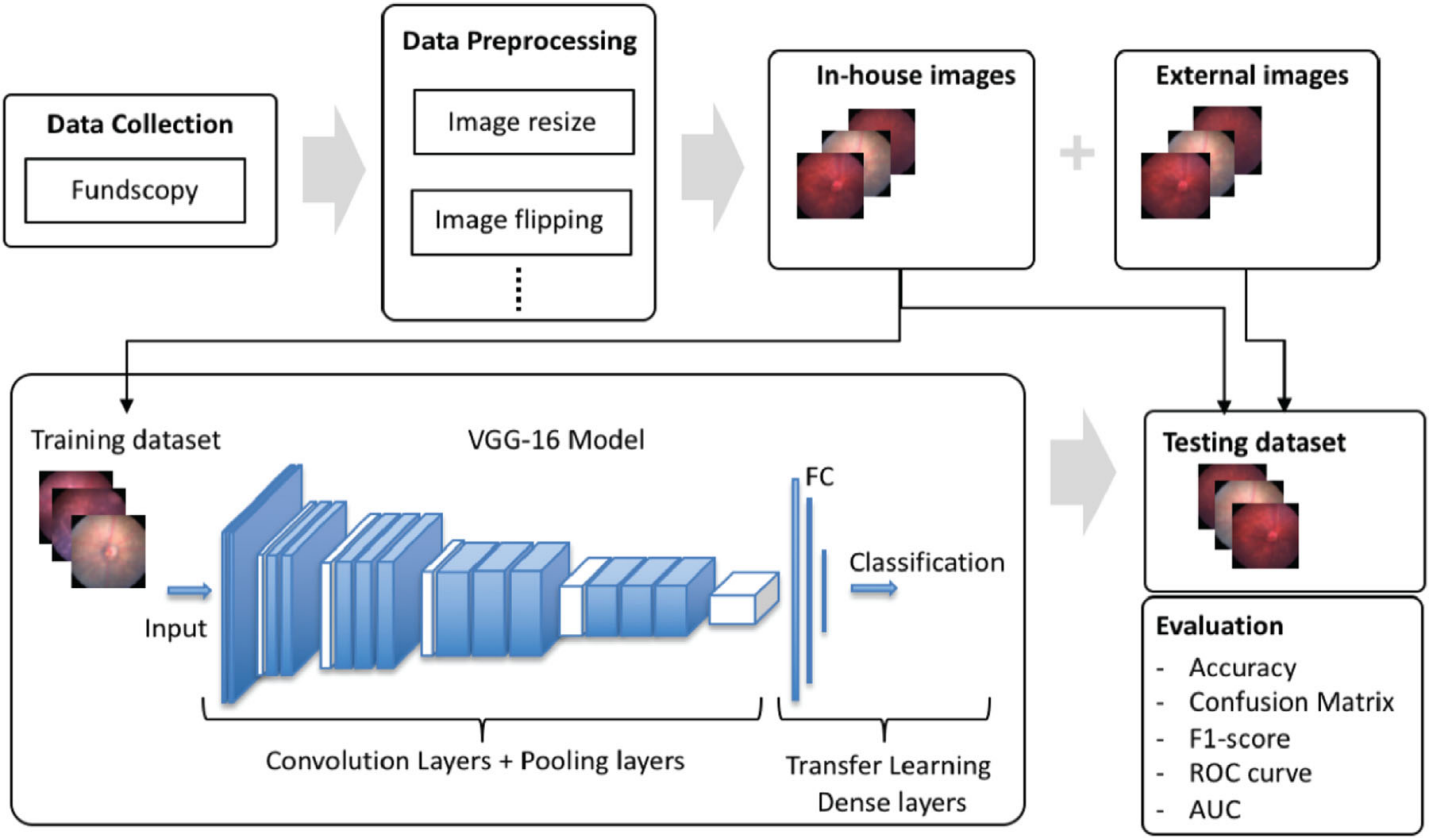
Workflow of our experimental design.

EAU was induced by active immunization with 150 µg bovine interphotoreceptor retinoid-binding protein (IRBP) and 300 µg human IRBP peptide, amino acid residues 1 to 20 (IRBP1–20) in a 0.2-mL emulsion 1:1 v/v with Complete Freund's Adjuvant containing *Mycobacterium tuberculosis* strain H37Ra (2.5 mg/mL). Mice also received *Bordetella pertussis* toxin (1 µg/mouse) concurrent with immunization. Mice were matched by age and sex, and for most experiments 6- to 8-week-old mice were used (14 mice per group). Clinical disease was established and scored by fundoscopy as described previously. Mice were maintained and used in accordance with National Eye Institute, National Institutes of Health Animal Care and Use Committee (ACUC) guidelines (Animal Study Proposals EY000262-19 and EY000372-14) and the study protocol was approved by the ACUC. The study relied on three images datasets to build the classifiers: in-house dataset, independent dataset, and external dataset (Fig. 1; Supplementary Table S1).

**Figure 2.**
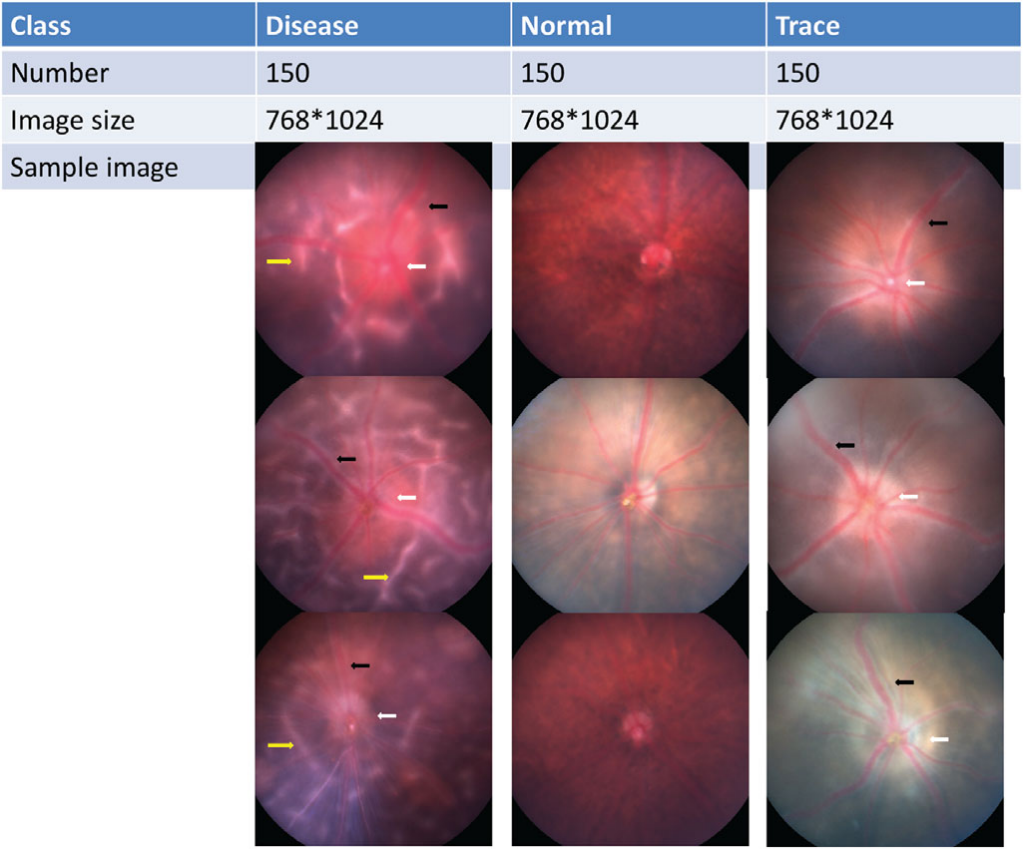
In-house datasets for C57B6 mice were used for the study. Fundus images were collected 14 days after immunization. Images were labeled as normal, trace, or disease. We selected 1500 images for model training, validation, and testing. An independent dataset of 180 images and an external dataset of 33 images were used for additional testing. *White arrow*, optic disc edema; *black* *arrow*, vasculitis; *yellow* *arrow*, retina folds.

The in-house dataset included 1500 color fundus images captured by Micron III fundoscopy (Phoenix Technology Group, Pleasanton, CA). Two animal experimentalists independently labeled each fundus image, and disagreements were resolved through discussion and input from the third animal experimentalist. Several sample images are shown in Figure 2. The normal class refers to images with no obvious lesion, whereas the trace class corresponds to images with optic disc edema and/or vasculitis. The disease class includes optic disc edema and vasculitis plus retina folds. The disease class was essentially assigned based on lesion size and location (Fig. 2).

The independent dataset included 180 fundus images that were annotated similarly to the in-house dataset but was kept aside for independent validation. The original resolution of images in these two datasets was 1024 × 768 pixels. Finally, the external dataset contained 33 color fundus images obtained from published EAU-related^18^^–^^21^ research papers by scanning or copying and digitizing images. Supplementary Figure S1 shows retesting (validation) in the independent subset, and Supplementary Figure S2 demonstrates retesting (validation) of the model using the external dataset. Some of the fundus images will be available upon request (dlvision4us@gmail.com).

### Overall Model

We first established a data analytics workflow before applying the CNNs in order to automatically perform an end-to-end process (Fig. 1). The data analytics workflow was comprised of the following tasks:

1. Data preprocessing
2. Model building and training
3. Model evaluations and interpretation

Unlike conventional machine learning models, CNNs do not require any human feature engineering efforts or intervention, as they take advantage of the hierarchical pattern in images and automatically assemble more complex patterns using smaller and simpler visual patterns. This independence from prior knowledge of the disease visual patterns is a major advantage that is key to eliminating the need for human effort and expertise to describe the disease in terms of its visual features. More details on the CNNs are provided in the next section.

### Data Preprocessing

Because the original resolution of these images varied, all images were preprocessed and resized to a fixed resolution of 224 × 224 pixels. The in-house dataset (1500 images) was split into training, validation, and testing datasets at the ratio of 8:1:1 to build the model; the independent and external datasets were used only for testing. To make the model robust to withstand any spatial variation in fundus images and to reduce the likelihood of overfitting while making the model more generalizable to new samples, we performed data augmentation, which substantially increased the size of the training dataset, as well. Specifically, randomized horizontal flip, vertical flip, 180° rotation, left skew, right skew, and zoom (1.5, 1.5) were applied to images (Supplementary Fig. S3). The new augmented subset included 1800 images, 600 per class.

### CNN Model for Disease Classification

CNNs have been commonly applied to image classification problems. It has been demonstrated that CNNs are powerful machine learning models that can extract a pool of features from images at different resolutions and perform successful classification tasks.^22^ A number of state-of-the-art CNN architectures have been published in the past decade, such as VGG,^23^ Inception,^24^ and MobileNet,^23^^,^^25^^,^^26^ and have been successfully adapted to medical analysis and applications.^27^ While developing effective CNN architectures to handle small subsets, we utilized appropriate numbers of samples to train the CNN model. We used a deep CNN architecture based on VGG-16,^23^ and we then adapted the VGG-16 pretrained model to solve our classification problem. The VGG-16, a state-of-the-art deep neural network, has achieved 92.7% top-five test accuracy in the ImageNet^28^ dataset of over 14 million images. In the VGG-16, images have been downsampled to a fixed resolution of 224 × 224 pixels and mapped to 1000 classes.

Supplementary Figure S4 depicts the proposed CNN. The inputs to the CNN model are fundus images; the output layer contains neurons with a Softmax activation function to produce a probability distribution for three classes of normal, trace, and disease. The deep convolutional layers of the pretrained VGG-16 model were used as powerful feature extractors, and the dense and Softmax layers that gave rise to the three classes were added and retrained using our EAU fundus images (Supplementary Fig. S4).

### Model Training and Prediction

Multiple versions of the CNN model were generated by fine tuning and adjusting various hyperparameters. More specifically, batch normalization was used with batch sizes set as 10. The model was trained on a graphics processing unit for 50 epochs, and the best model (monitored based on accuracy) was selected. Adam optimization was used, and the initial learning rate was set at 0.001 (Supplementary Fig. S5). Weights of the deep convolutional layer of the VGG-16 pretrained model were frozen, and the weights in dense layers were updated during the training process.

The categorical cross entropy is used as a loss function for the multiclassification and is expressed as

(1) $$L\left(y,\hat{y}\right)=-ylog\hat{y}-\left(1-y\right)\log\left(1-\hat{y}\right)$$

The cost function of the total training images is expressed as

(2) $$\begin{array}{c} J\left(w,b\right)=-\frac{1}{m}\sum_{i=1}^{m}[(y^{\left(i\right)}log{\hat{y}}^{\left(i\right)}\\ +\left(1-y^{\left(i\right)}\right)\log\left(1-log{\hat{y}}^{\left(i\right)}\right)] \end{array}$$

where *y* is the true label of the image, and $\hat{y}$ is the model predicted label.

Confusion matrices and AUCs were used to evaluate the models. We also computed the specificity and sensitivity of the models with regard to detecting normal and EAU. Specificity and sensitivity are defined as

$$Specificity=\frac{TP}{TP+FP}$$

$$Sensitivity=\frac{TN}{TN+FN}$$

Each image from the test set was resized to 3 × 224 × 224 pixels and then reshaped to a one-dimensional vector of size (1, 3 × 224 × 224). The whole test set was transformed to a matrix of size (150, 3 × 224 × 224), and the pixel values were rescaled to the range of (0, 1). Principal components analysis (PCA) and *t*-distributed stochastic neighbor embedding (t-SNE) were performed on the whole test set, and three major components were used to visualize the distribution in three dimensions.

## Model Evaluation Results

### Classification of EAU Severity Based on Fundus Images

**Figure 3.**
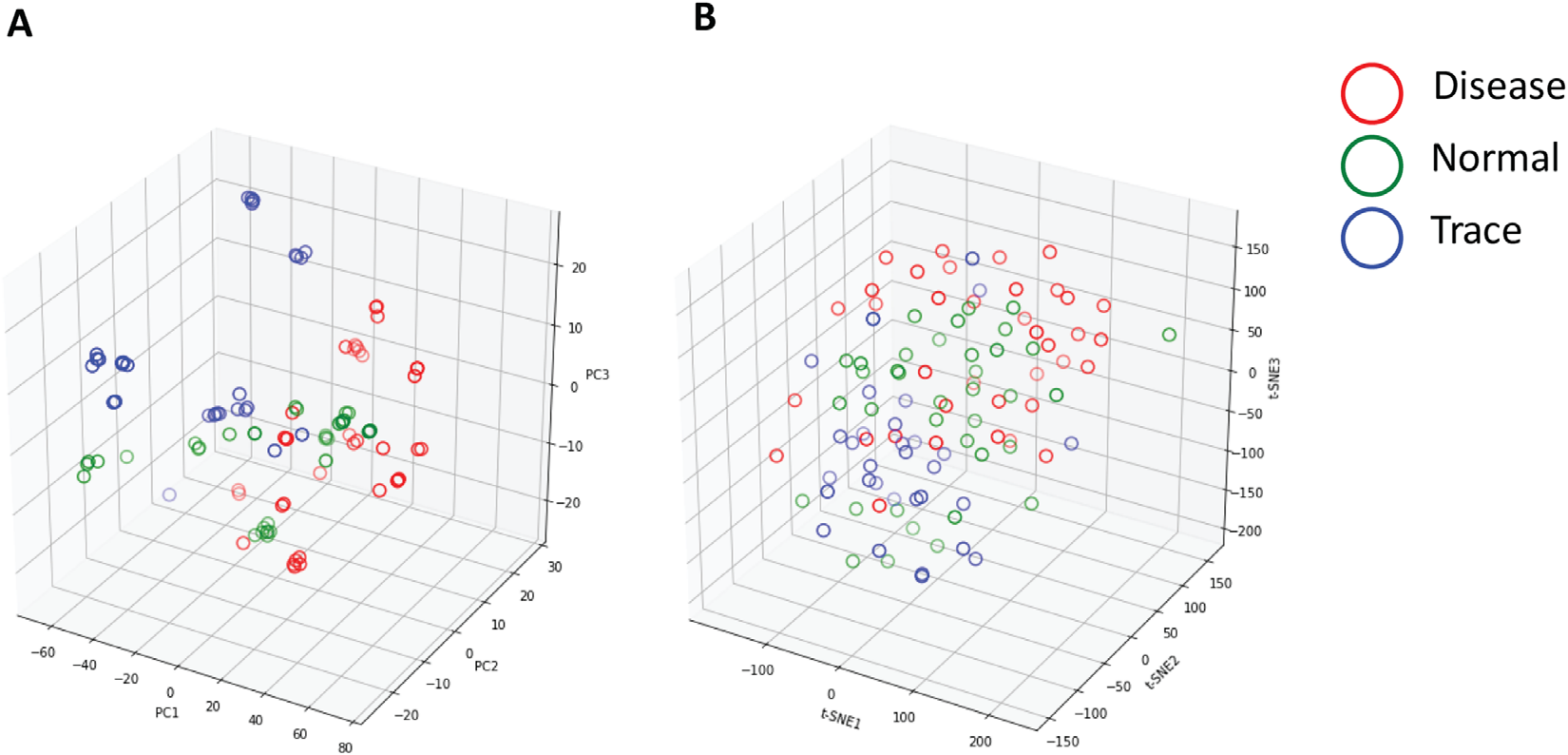
Visualization of the in-house testing dataset in a three-dimensional space. (**A**) Visualization by PCA, and (**B**) visualization by t-SNE. Each *circle* represents a fundus image.

In reference to the workflow illustrated in Figure 1 and the data preprocessing section, the in-house dataset included 1500 mouse fundus images captured from naive mice or EAU disease mice (Fig. 2) and equally distributed into 500 images in each class: normal, trace, or disease. To identify the degree of overlap of fundus images in the original space, we first applied PCA and t-SNE to visualize the images in the testing dataset. We found a significant degree of overlap among images from the three groups (Fig. 3). This demonstrated that features from original fundus images without CNNs were not successfully captured by linear PCA or nonlinear t-SNE. We then trained the CNN models to learn the underlying features of those images to identify EAU.

**Figure 4.**
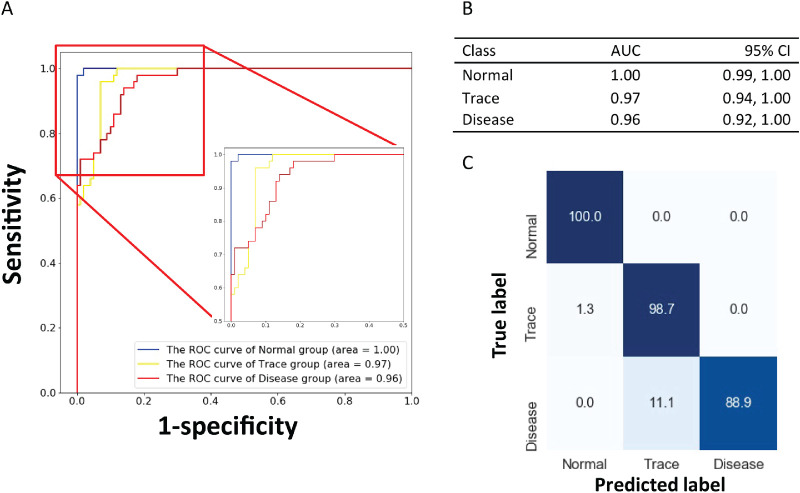
Evaluation of the CNN model. (**A**) The model was evaluated on the in-house testing dataset (150 images). (**B**) AUCs and 95% CIs were calculated for each class. (**C**) Confusion matrix for the in-house test dataset; numbers represent percentages.

Figure 4 shows the receiver operating curve (ROC) of the in-house testing dataset. The AUCs were 1.00 (95% confidence interval [CI], 0.99–1.00), 0.97 (95% CI, 0.94–1.00), and 0.96 (95% CI, 0.92–0.99) for the normal, trace, and disease classes, respectively (Fig. 4). Sensitivity values were 0.98, 0.92, and 0.84 for the normal, trace, and disease classes, respectively, and specificity values were 1.00, 0.91, and 0.96 for the normal, trace, and disease classes, respectively (Table 1). We examined the images that were misclassified and observed that the highest confusion occurred between normal and trace or between trace and disease (Fig. 4C); however, the model was able to classify all normal occurrences correctly. Therefore, the late stage of EAU can be successfully and accurately detected by the proposed model.

**Table 1.** Model Evaluation Metrics on the In-House Testing Dataset

Class	Sensitivity	Specificity
Normal	0.98	1.00
Trace	0.92	0.91
Disease	0.84	0.96

A total of 150 testing images (50 per class) were used for the model evaluation. See Figure 4.

To ensure the generalizability and robustness of the model for detecting EAU, out of the initial training, testing, and validation data we retested the model on a subset of images collected separately after developing the model. This independent testing dataset included a total of 180 images with 60 images from each group. The weighted AUC of the model on this subset initially indicated a high degree of generalizability for the independent data. To further assess the developed model on a dataset that was not collected in our laboratory, we collected an external dataset comprised of images published in the literature on the same subject. The external dataset included 33 EAU images with average resolution significantly lower than our in-house and independent datasets (Supplementary Fig. S6). Nevertheless, the AUC of the model using this subset was approximately 0.90. The detailed statistics of the model performance are summarized in Tables 2 and 3.

**Table 2.** Model Evaluation Metrics on the Independent Testing Dataset

Class	AUC	95% CI
Normal	1.00	0.99–1.00
Trace	0.97	0.94–1.00
Disease	0.96	0.90–1.00

A total of 180 testing images (60 per class) were used for the model evaluation.

**Table 3.** Model Evaluation Metrics on the External Testing Dataset

Class	AUC	95% CI
Normal	0.99	0.95–1.00
Trace	0.88	0.74–1.00
Disease	0.90	0.76–1.00

A total of 33 testing images (11 per class) were used for the model evaluation.

We compared the performance of our CNN model with that of human experts. Two human experts in EAU had annotated our independent testing dataset with 180 images into the three classes of normal, trace, or disease. Their overall accuracy rates were 0.93 and 0.94, respectively. The kappa score was 0.86 (95% CI, 0.81–0.91) (Supplementary Table S2).

### Interpretability of the CNN Model

**Figure 5.**
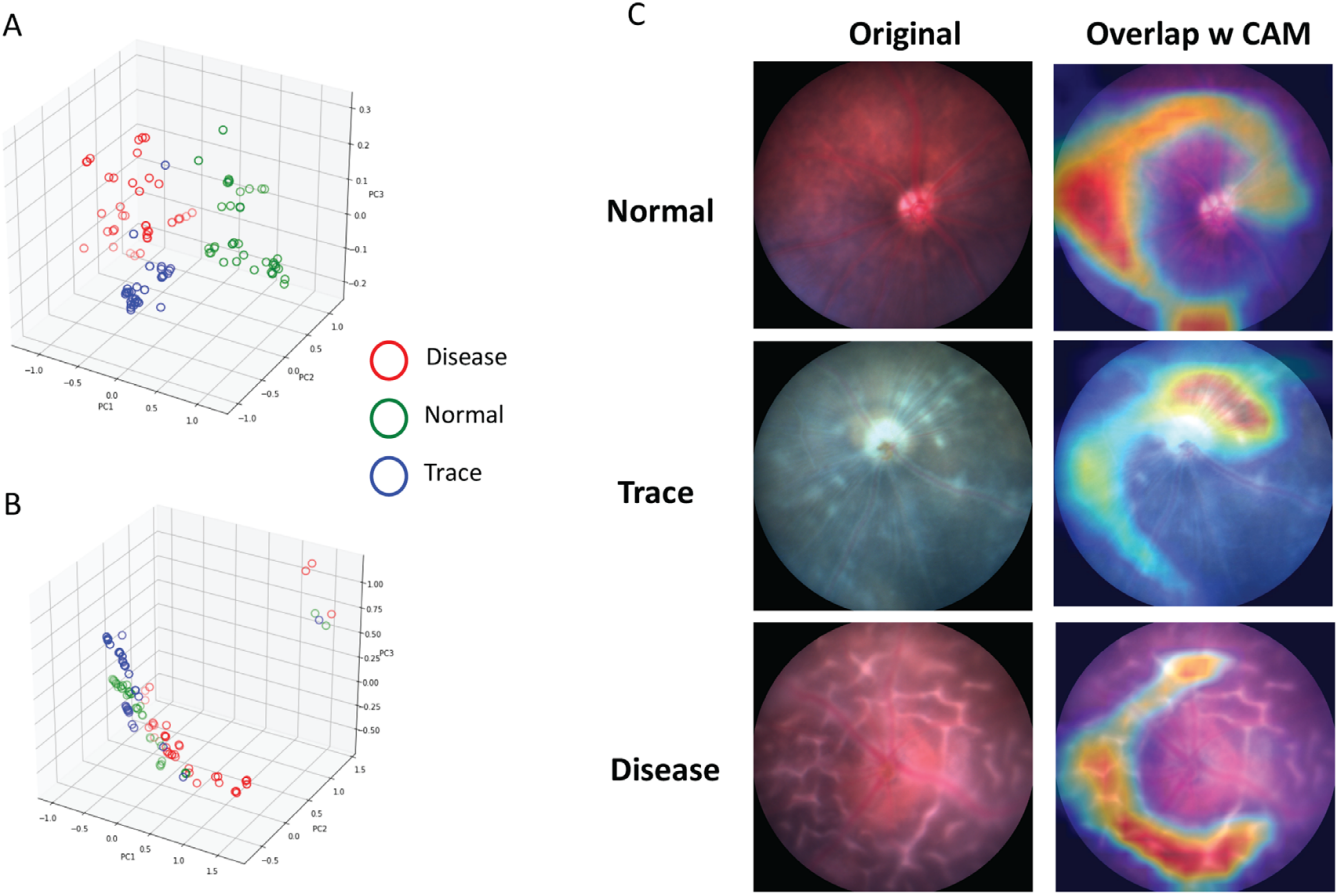
Interpretability of the CNN model based on the in-house testing dataset. (**A**) PCA visualization of feature representations from the output of the first dense layer. (**B**) PCA visualization of feature representations from the output of the last convolution layer. Each *circle* represents a fundus image. (**C**) Class activation map for the output of the last convolution layer.

To illuminate the black-box nature of the deep learning models and explain findings, we implemented two approaches. To directly analyze image features transformed by our model, we used PCA to visualize the output feature representations of each layer. The single dense layer clearly separated our testing dataset into three distinct groups, and the pattern was consistent with outcome of the deep learning model (Fig. 5A); in contrast, convolution layers do not have obvious capacity of class separation (Fig. 5B). To understand which parts of the retina (area on the fundus images) contributes the most to the deep learning class assignment (output probability distributions), we generated gradient-weighted class activation maps (Grad-CAMs) corresponding to different convolution layers.^29^ The feature map generated by the last convolutional layer was directly used to calculate the probability distribution. By overlaying Grad-CAMs with the original images, we found that the last convolution layer was primarily looking at three retinal regions (areas on the fundus image): optic disc, blood vessels, and periphery fundus areas (Fig. 5C). This is quite similar to the clinical manifestation of EAU and in line with the grading process executed by human experts. Therefore, our model not only achieved a high accuracy in identifying EAU but also provided outcome with clinical relevance.

## Discussions

In this study, we proposed a deep learning model to automatically detect disease features in mouse retina. We have used the mouse EAU model to demonstrate that a well-designed deep learning models can assist clinicians and other experts in consistently making accurate and unbiased diagnoses of the severity of uveitis. Our CNN model achieved an average AUC of 0.98 using 1200 fundus images for training. To ensure generalizability, we retested the model on an independent subset of images that were collected after we developed the model, and we achieved a similar AUC of 0.98. More strikingly, our model was able to classify images used in EAU publications with an AUC of over 0.90.^18^^–^^21^ In our analysis, we built numerous AI models, including VGG-16, Inception-v3, and MobileNetV2, but we achieved the best accuracy by using a VGG-16-based transfer learning model. To our knowledge, this is the first model developed for studying EAU. Our model achieved slightly better performance than human experts, and eliminated inter- and intra-observer variations.

A number of deep-learning-based image classification models have been developed for clinical use on human patients,^30^ because deep learning models have shown substantial advantages in at least three aspects: (1) consistency in eliminating inter- and intra-observer variations; (2) accuracy in successfully learning complex features and patterns at various resolutions to achieve a high rate of accuracy, whereas human experts rely on experience and extensive training; and (3) cost-effectiveness in reducing the tedious and costly process of annotations by human experts. Although they typically apply to clinical research and practice, the same justifications can be applied to experimental animal research, as well. The application of deep learning to experimental animal research can lead to standardization in the interpretation of results across different laboratories, allowing for more accurate comparisons and collaborations. This is important, as it is currently quite challenging to compare the results of the various research facilities that have performed testing and interpretation under a multitude of conditions with variable levels of skill in categorizing uveitis. Therefore, deep learning is a plausible technique for promoting the reproducibility and standardization of animal research; however, deep-learning-based tools are not yet readily accessible to animal researchers. Our current work is an effort to fill this gap.

### Data Collection for Transfer Learning and Training the CNN Model

The training of initial deep learning models normally requires thousands to millions of labeled images.^31^ The quality of such datasets is critical and significantly determines the outcome of the trained model; however, transfer learning can keep millions of parameters intact for different deep learning applications. As such, in a new application, only a subset of those previously identified parameters along with hyperparameters can be fine tuned, which requires significantly smaller datasets. Based on these advantages, we chose to take advantage of transfer learning applied to subsets of mouse model to optimize our procedure.

### Data Collection for Validating the CNN Model

Although the overall aim is to collect the most representative images and minimize the effect of various background noise, such as light reflection artifacts, achieving this goal is challenging in real-world applications. As such, we investigated the accuracy of the proposed models that were trained with in-house datasets (generated in our lab) with external datasets that were collected from other facilities across the world. This subset had significantly lower quality and differing focusing planes, fields of view, angles of view, background colors, and image contrasts compared to our in-house dataset. This diverse external dataset with diverse image quality proved to be critical for the training process in order to achieve high accuracy and generalizability. This approach is not practical for image collection on human subjects, as most human images are retrospectively collected from hospital or healthcare databases. From this perspective, implementation of deep learning models in animal research offers a unique advantage in terms of data collection.

### Accuracy

The accuracy of our model based on the independent dataset that was collected after developing the model was on par with the accuracy based on the in-house dataset. It is worth noting that the accuracy of the model based on the external dataset was lower than the accuracy of the model based on the in-house or independent datasets. This could be for a variety reasons. First, the external dataset included images with significantly different machine and camera settings; for example, artifacts such as light reflection introduced during imaging could easily confuse the model. Second, the external dataset was collected by cropping and pasting images from published papers in the literature with different resolutions ranging from 100 × 97 pixels to 219 × 219 pixels, which is significantly lower than in-house datasets. For some of the external images the resolution was even lower than the input image resolution required for deep learning. Based on the confidence level of our model for each image, we determined that the resolution significantly impacted the accuracy. Even taking into account the effects of varying resolution, the proposed model overall provides high accuracy and offers generalizability on new images captured under varying conditions. Such robustness is highly important for integrating AI models into vision research and eventually into clinical practice.

### Interpretability

Although deep learning models have received significant interest over the past few years, their black-box nature limits interpretation, particularly in healthcare applications. Essentially, interpretability is one of major technical obstacles in the implementation of deep learning. We utilized two different approaches to illuminate this black box to enhance the interpretability of the proposed AI models. First, PCA analysis revealed that without deep learning the model would not reach the high level of accuracy we obtained, due to the high overlap between samples from different classes. Supplemental PCA also revealed that the single dense layer was sufficient to achieve high accuracy in recognizing different features within the dataset. Second, and clinically more important, by using Grad-CAMs we found that deep convolution layers are able to extract hidden retinal features that significantly drive deep learning prediction. More specifically, we determined that the most important retinal regions for deep learning identification/classification of EAU included blood vessels, the optic disc, and retinal periphery regions, all of which correspond to the regions used by human experts to identify and classify EAU. Thus, deep learning results, rather than representing a black box result, may reveal important links underlying disease, which suggests further potential for clinical relevance.

### Limitations of the Present Study

Our current model classifies the state of disease based on the whole fundus image. It would be desirable to train the model to recognize individual disease features and give an overall score by considering individual features. However, this is a limiting factor for all AI models and not limited to our study. The major misclassification error of our model was observed in the uncertainty between trace and disease classes; however, this is a major problem for human experts, too. In fact, disease is a continuum, and distinguishing a threshold to separate different severity levels is very challenging even for highly trained and skilled human experts. Therefore, the role of high-quality ground-truth labels is critical for the model performance, which is reasonable given the nature of supervised learning. Follow-up studies with greater numbers of images and greater numbers of human experts annotating the images are desirable to reduce the effect of this limitation.

Our current model classifies only three categories, but classification into three categories provides a better means to reach a consensus among human graders compared to classification into only two (normal vs. abnormal) classes, thus offering better potential for being integrated into real-world applications. Nevertheless, in animal models we usually need more categories corresponding to different severity levels and clinical scores; therefore, datasets with larger numbers of severity levels are desirable for developing models with better real-world applications.

In the past few years, the generative adversarial network has emerged as a powerful method for generating more training data.^32^^,^^33^ This alternative approach to generating data could address the issue of the limited number of training samples in most applications. Although a larger training dataset may result in a better classification performance in general, our study demonstrates that high accuracy of deep learning models can also be achieved using transfer learning and a mid-size training dataset. As a next step, models such as ours will require independent and external validations to ensure their generalizability.

## Supplementary Material

Supplement 1

Supplement 2

Supplement 3

Supplement 4

Supplement 5

Supplement 6

Supplement 7

Supplement 8
