# Supplementary figures and images for: Identifying Mouse Autoimmune Uveitis from Fundus Photographs Using Deep Learning
Source: Transl Vis Sci Technol. 2020 Dec 2;9(2):59. doi: 10.1167/tvst.9.2.59 (PMC7718814; doi:10.1167/tvst.9.2.59)

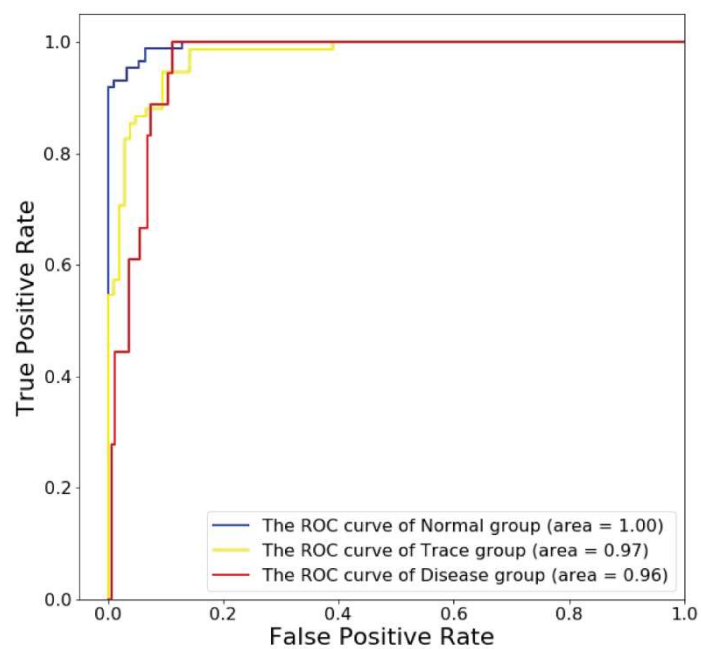

**FigS1.** Re-testing with independent dataset

Supplement: Supplement 1 [file tvst-9-2-59_s001.pdf]

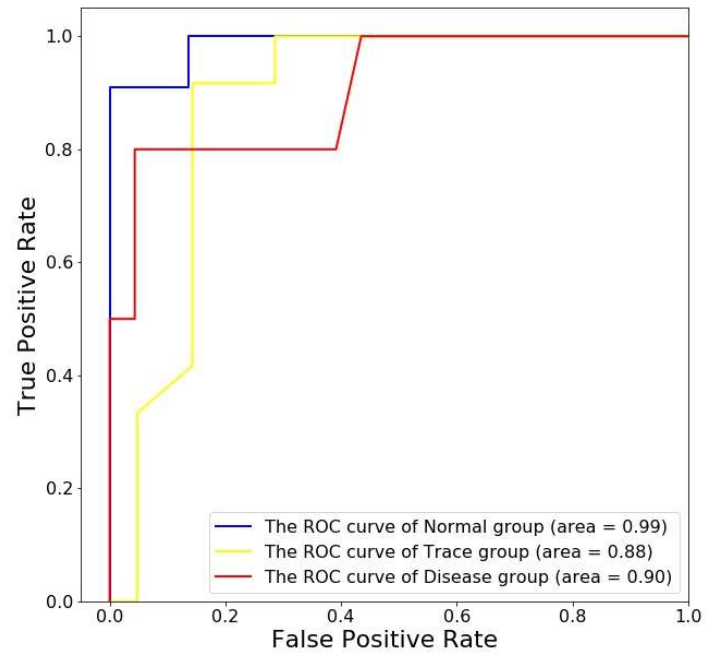

**FigS2.** Re-testing with external dataset

Supplement: Supplement 2 [file tvst-9-2-59_s002.pdf]

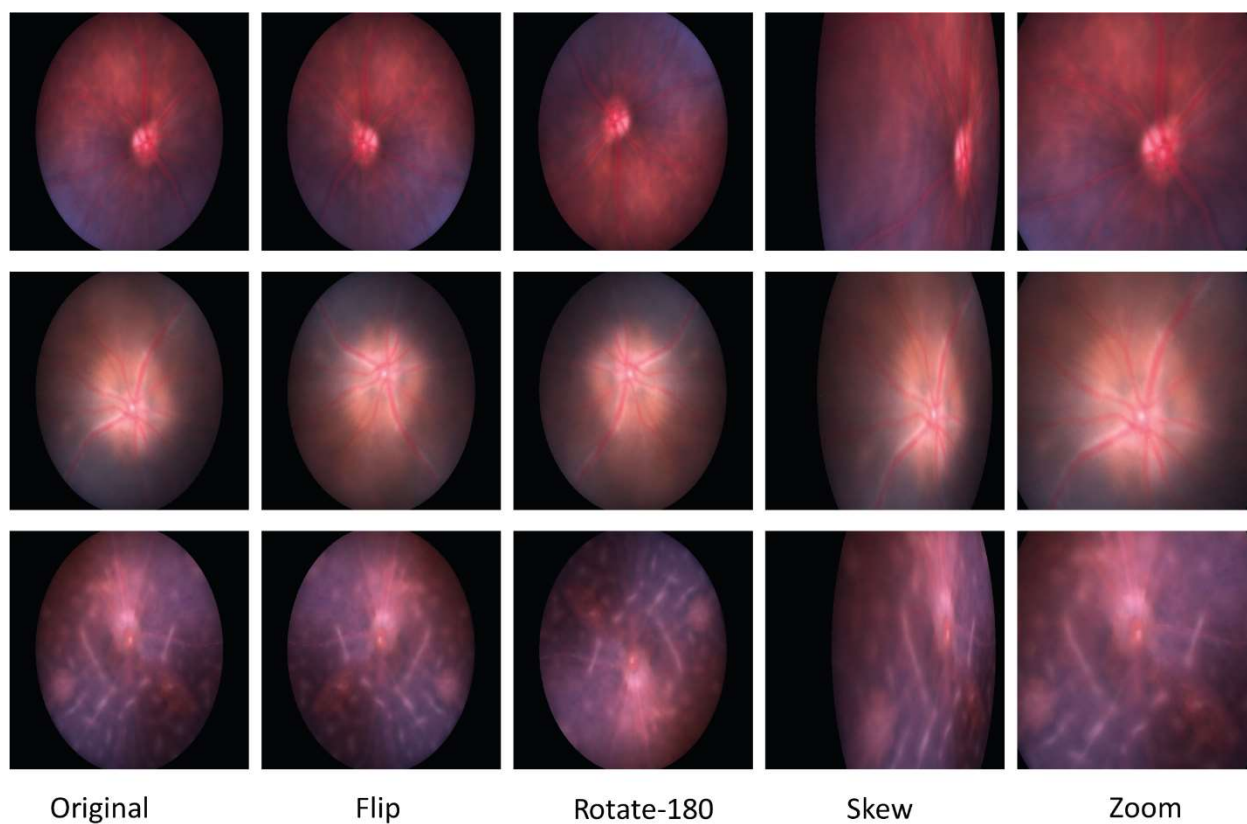

**FigS3.** Image Augmentation

Supplement: Supplement 3 [file tvst-9-2-59_s003.pdf]

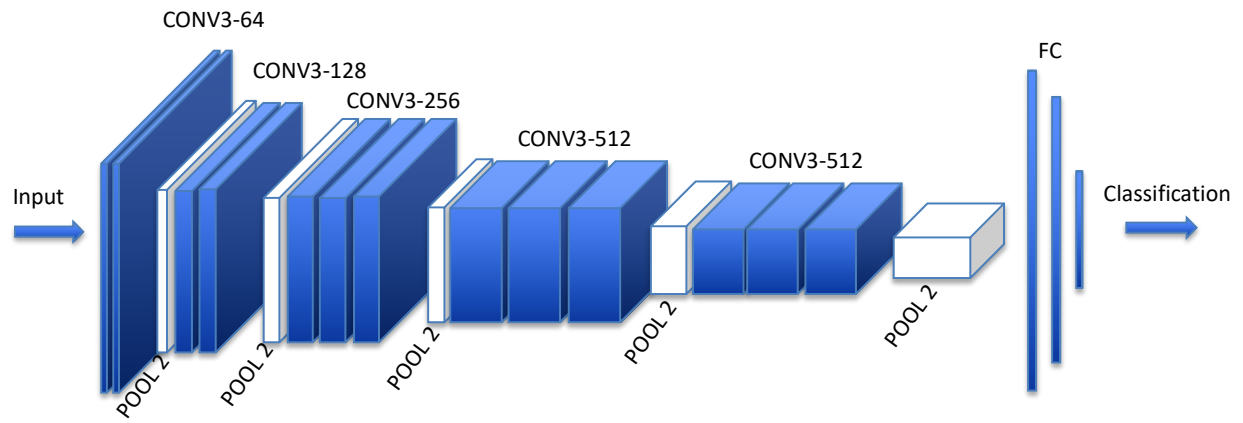

**FigS4.** Model architecture

Supplement: Supplement 4 [file tvst-9-2-59_s004.pdf]

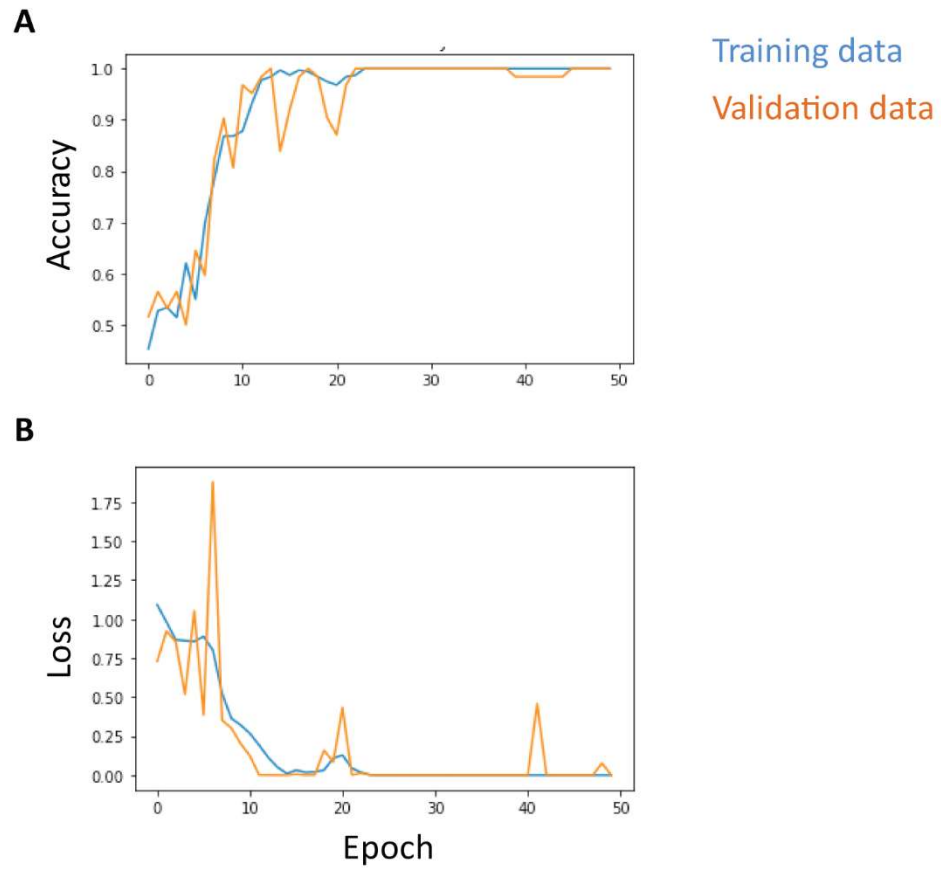

**FigS5.** Plots of training accuracy and loss

Supplement: Supplement 5 [file tvst-9-2-59_s005.pdf]

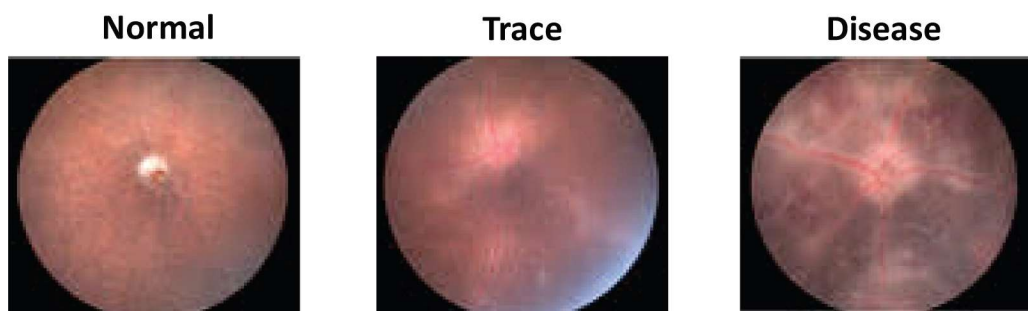

**FigS6.** Examples of external dataset

Supplement: Supplement 6 [file tvst-9-2-59_s006.pdf]
