# Supplementary material for: Identifying Mouse Autoimmune Uveitis from Fundus Photographs Using Deep Learning
Source: Transl Vis Sci Technol. 2020 Dec 2;9(2):59. doi: 10.1167/tvst.9.2.59 (PMC7718814; doi:10.1167/tvst.9.2.59)
Supplement: Supplement 7 [file tvst-9-2-59_s007.pdf]

Table S1 Datasets and the corresponding number of images

Datasets		Number of images
In-house dataset	Training	1200
	Validation	150
	Testing	150
Independent testing dataset		180
External testing dataset		33
