# Supplementary material for: Identifying Mouse Autoimmune Uveitis from Fundus Photographs Using Deep Learning
Source: Transl Vis Sci Technol. 2020 Dec 2;9(2):59. doi: 10.1167/tvst.9.2.59 (PMC7718814; doi:10.1167/tvst.9.2.59)
Supplement: Supplement 8 [file tvst-9-2-59_s008.pdf]

Table S2 Performance of human graders on independent testing dataset (180 images)

	Accuracy	Kappa (95% CI)
Grader A	0.93	0.86 (0.81-0.91)
Grader B	0.94	
